# Rheology of Emulsion-Filled Gels Applied to the Development of Food Materials

**DOI:** 10.3390/gels2030022

**Published:** 2016-08-16

**Authors:** Ivana M. Geremias-Andrade, Nayla P.B.G. Souki, Izabel C.F. Moraes, Samantha C. Pinho

**Affiliations:** Department of Food Engineering, School of Animal Science and Food Engineering, University of São Paulo (USP), Av. Duque de Caxias Norte 225, Jd. Elite, Pirassununga, Sao Paulo 13635-900, Brazil; ivana.geremias@usp.br (I.M.G.-A.); nayla.souki@usp.br (N.P.B.G.S.); bel@usp.br (I.C.F.M.)

**Keywords:** emulsion-filled gels, rheology of gels, rheological modeling, emulsion gels, emulgels

## Abstract

Emulsion-filled gels are classified as soft solid materials and are complex colloids formed by matrices of polymeric gels into which emulsion droplets are incorporated. Several structural aspects of these gels have been studied in the past few years, including their applications in food, which is the focus of this review. Knowledge of the rheological behavior of emulsion-filled gels is extremely important because it can measure interferences promoted by droplets or particle inclusion on the textural properties of the gelled systems. Dynamic oscillatory tests, more specifically, small amplitude oscillatory shear, creep-recovery tests, and large deformation experiments, are discussed in this review as techniques present in the literature to characterize rheological behavior of emulsion-filled gels. Moreover, the correlation of mechanical properties with sensory aspects of emulsion-filled gels appearing in recent studies is discussed, demonstrating the applicability of these parameters in understanding mastication processes.

## 1. Introduction

Obesity has become a public health problem in several countries, which has led the food industry to engage in developing new products with desirable attributes of taste and texture without the addition of high amounts of fat, sugars, and salt [1]. The consumption of trans fats and saturated fats has been associated not only with obesity but also with high blood levels of cholesterol, cancer, and heart diseases [2,3,4]. Consequently, consumers have started to demand highly nutritious foods containing beneficial compounds to health that could prevent these diseases [5].

Dairy products such as ice creams, yogurt, and cheese, as well as processed meat products, feature a high content of saturated fat and can be described as emulsion-filled gels with a high content of saturated long-chain fatty acids. An overall reduction of fat content in foods can impact their sensory, structural, and rheological properties. On the other hand, lipids have an important role as vitamins and essential fatty acids carriers [6]. Furthermore, the emulsified fats exert a favorable impact on the texture and palatability of these products [7].

Emulsion-filled gels are matrices of polymeric gels (proteins or/and polysaccharides) into which emulsion droplets are incorporated. These structures are classified as soft solids and can be produced from a stable emulsion incorporated into the gelled continuous phase [6,8]. In the current literature, these products are often described as “emulsion-filled gels”, “composite gels” [9,10] or “emulsion gels” [8]. According to Dickinson [8], an emulsion-filled gel presents hardness higher than that presented by an equivalent protein gel, which is an indicator of the strong reinforcement provided by the emulsion oil droplets to the semi-solid.

A great advantage of the application of emulsion-filled gels in food formulations is that these structures can assist in the production of food with reduced fat content. The combination of gelling agents and oils for obtaining emulsion-filled gels is considered as highly effective in achieving this aim. Both proteins and polysaccharides have been extensively tested in the production of emulsion-filled gels [10,11].

An extremely important aspect of the emulsion-filled gels is their rheological behavior. The textural properties of these systems are strongly affected by the inclusion of emulsion droplets, which influence the texture characteristics and improve the creaminess perception of the product [7,12]. For instance, the addition of oils emulsified with milk proteins can reinforce the structural properties of milk protein gels due to strong interactions between the adsorbed proteins on the surface of the oil droplets and the protein gel matrix [13,14,15]. Therefore, obtaining information about the rheology of emulsion-filled gels is an important analytical method that promotes the understanding of the structural organization and the interactions between food components [10].

This article reviews the rheological measurements of emulsion-filled gels focusing on food applications and how the inclusion of emulsion and their particles/droplets affects these systems. We discuss how and why rheological methods can be useful to predict the behavior of emulsion-filled gels when the filler characteristics are changed. Finally, a discussion about sensorial perception of these products and its correlation with rheological characterization has been provided.

## 2. Definition and Characteristics of Emulsion-Filled Gels 

**Figure 1:**
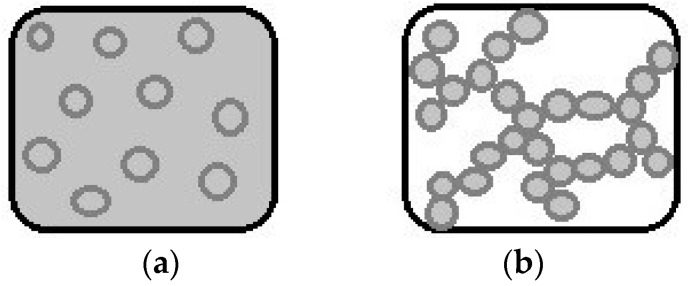
Theoretical representation of the two structures involving gels and emulsions in combination: (**a**) Emulsion-filled gel and (**b**) Emulsion gel.

Emulsion-filled gels are defined as a complex colloidal material formed by the combination of an emulsion dispersion and a gel phase. The combination of these two systems can be divided into two distinct structures (Figure 1): (a) emulsion-filled gels and (b) emulsion gels, in which the major studies of this complex colloidal system have been conducted with proteins. However, some recent studies have used polysaccharides in the production of gels. The emulsion-filled gels consist of the substitution (partial or total) of the water of a gel matrix by an emulsion, where the particles of emulsion are incorporated in this matrix and the materials have solid-like rheological properties. The properties are determined predominantly by the network properties of the spatially continuous matrix. However, emulsion gels are particulate gels whose rheological behavior is determined by the properties of the network containing the aggregated emulsion droplets. These two arrangements are theoretically distinct, but in practice, an emulsion-filled gel consists of a hybrid network comprising incorporated droplets and partially aggregated droplets, simultaneously [8].

**Figure 2:**
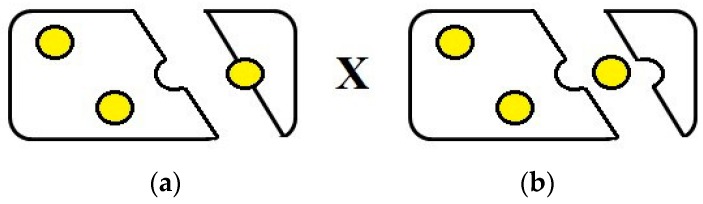
Schematic representation to differentiate the two filler particles: (**a**) Active particles and (**b**) Inactive particles.

The mechanical properties of emulsion-filled gels depend on the physicochemical properties of the gel matrix and the emulsion droplets, the size, the volume fraction, and the distribution of the emulsion droplets, as well as the strength and the interactions among the emulsion droplets and the gel matrix [16]. The interactions depend on the properties of the surface of emulsion droplets due to the different properties of the surfactants used to produce the emulsions [17]. The nature of the interactions determines if the droplets are, or are not, bound to the gel network. If the particles are mechanically connected to the matrix, they are named as active particles and, if not, as inactive particles (Figure 2) [16,18]. The active filler particles are bound to the gel matrix due to the interactions occurring among the macromolecules of the gel matrix and the surfactant molecules, and they can increase or decrease the stiffness of the gel. The inactive filler particles are not bound to the gel matrix and have low chemical and physical affinity with the macromolecules of the matrix, while always causing a reduction of stiffness [16,19].

The emulsion produced for subsequent incorporation into the biopolymer gel has different characteristics due to the types and concentrations of lipids and surfactants used as well as the methods of production. After the production of the emulsion, the next step is its incorporation into a biopolymer matrix (protein and polysaccharides, in food applications) for the production of an emulsion-filled gel. This step involves gelling of the continuous phase or/and can occur by aggregation of the emulsion droplets [8].

The literature presents several studies on the formation of emulsion-filled gels from proteins, such as whey protein isolate, gelatin, and soy protein isolate [6,17,19,20,21,22,23]. Studies related to the production of gels from proteins have used production methods such as heat treatment, acidification, enzyme treatment, and cold induction by the addition of calcium salts [8]. Other authors have studied the formation of emulsion-filled gels from polysaccharides such as gellan [10], inulin [11], κ-carrageenan [24], alginate [25], agar [26] and others. Generally, emulsion-filled gels of polysaccharide as gelling agent are produced by homogenization, and, depending on the polysaccharide, the presence of some ions (like Ca^2+^) is required. Paradiso et al. [11] used three different homogenization mechanisms (mechanical, ultrasonic, and cold ultrasonic) for the production of emulsion-filled gels using inulin as gelling agent. Lorenzo et al. [10] used mechanical homogenization of gellan gum gels filled by emulsion droplets. However, polyssacharides such as alginate and κ-carrageenan form gels in the presence of salts [25,26]. Despite production of gels by globular food proteins, they form gels when heated at a temperature of 70 °C and at sufficiently high concentrations. Gel formation occurs due to the denaturation and aggregation capacity of the proteins on heating the dispersions, and this capacity occurs because of the development of hydrophobic interactions between the exposed non-polar regions of the globular protein molecules. Heat treatment involves three steps, denaturation, aggregation, and gelling that occur simultaneously, and gel formation occurs by cross-linking of aggregated molecules [8,27].

Treatment by acidification is common in proteins that can self-associate and form gels when the pH value is reduced to a value next to the protein isoeletric point. This leads to intense repulsion among protein molecules, as well as to increase the degree of aggregation of the proteins, resulting in the formation of a three-dimensional gel network [28,29].

In enzyme-induced gelation, covalent protein cross-linking occurs by the addition of transglutaminase that catalyzes the acyl transfer reaction between lysine residues and glutaminyl residues [9,30,31]. The characteristics of the cross-links lead to the formation of a gel with different mechanical responses in comparison to that of heat-set or acid-set gels, whose rheological characteristics are more elastic [8,32].

Cold-set gels are obtained by addition of ions. Production of these gels involves preheating of the dispersion that causes denaturation of the protein, leading to aggregation without gelation. These aggregates are eventually responsible for the formation of the gel network after the subsequent addition of ions. Therefore, the denaturation and aggregation steps occur separately [27,33]. When salt is added to the cooled dispersion, it causes induction of cross-linking among the aggregated protein molecules, resulting in gel formation. The characteristics of gels such as texture, water-holding capacity, permeability, and appearance, as well as the spatial organization and structure, depend on the salt concentration used to induce gelation [33].

### Parameters that Impact the Rheological Behavior of Emulsion-Filled Gels

The gelled continuous phase is primarily responsible for the rheological characteristics of emulsion-filled gels [8,34]. Dickinson [8] stated that a viscoelastic behavior is observed in an oil-in-water emulsion when its continuous phase is formed by a viscoelastic biopolymer solution or gel. However, the inclusion of dispersed filler particles has a profound influence on the textural properties of gelled systems like emulsion-filled gels [12].

The presence of particles causes changes in the large deformation as well as the fracture properties of the gels, due to interactions among the particles (dispersed phase) and the gel matrix (gelled continuous phase) [34].

As per the classical van der Poel theory [35], the determinant factors for the influence of fillers in a gel matrix filled by an oil-in-water emulsion are the average particle size of oil droplets, their volume fraction, and their spatial distribution in the biopolymer matrix (single droplets or aggregated structures) of the fillers [17,21,34,35,36,37].

Van der Poel theory predicts the rheological behavior (more specifically, the shear modulus) of a gelled material filled by spherical particle dispersion distributed homogeneously. When the gel matrix is less rigid than the filler particles (*G*_filler_ > *G*_matrix_), the shear modulus (G) of the filled gel increases with increasing shear moduli of the filler particles and the matrix. Smith [38] simplified this theory and van Vliet [16] applied it to particle-filled gels. The limitations of this theory have been observed in systems where there is a crowding effect of filler particles at a high volume fraction and packing limit of particles [39] or if the emulsion droplets are flocculated [9,36]. Some studies have been conducted to develop models in which all these parameters are considered [39,40,41,42,43].

Some of the parameters that influence the emulsion-filled gels rheological properties are as follows: (i) the oil concentration and gelling material concentration [21,37]; (ii) the nature of filler–matrix interactions [34,37]; (iii) the physical state of the lipid (liquid or solid) [6,44]; (iv) the morphology of the gelled network [34]; and (v) the gel production process (cold-set, hot-set, acidification, and enzymatic), pH, and ionic strength [45,46,47].

The final gel strength is determined by the magnitude of the interactions (hydrogen bonds, hydrophobic interactions, electrostatic interactions, and covalent bonds) between different structural elements (biopolymer matrix–biopolymer matrix, biopolymer matrix–oil droplets, and oil droplets–oil droplets of the emulsion) [34,46]. The nature of the biopolymer matrix–oil droplet interaction depends on the interaction between the surfactant/emulsifiers for emulsion production and the gelling material [6,8,37]. A displacement of the protein from oil-in-water interfaces can occur as an action of small-molecule surfactants [8].

There are several studies investigating the impact of the aforementioned parameters on the rheological behavior of emulsion-filled gels. Dickinson and Chen [37] studied the impact of different emulsifiers (Tween 20 and whey proteins) on the rheological measurements of heat-set protein gels (whey protein) filled by oil-in-water (O/W) emulsions. According to the authors, when emulsions were stabilized by Tween 20, droplets did not interact with the gel matrix (inactive particle fillers). However, when the emulsions were stabilized by whey proteins, the droplets interacted with the gel matrix (active particle fillers). Whey protein can partially unfold in neutral pH and at temperatures >70 °C, and under these conditions, protein aggregation occurs, probably exposing the hydrophobic groups that interact with the protein network structure. Therefore, the whey protein-stabilized oil droplets increase the gel strength, whereas the oil droplets stabilized by Tween 20 weaken the gel matrix.

Van Vliet [16] studied the interaction between particles and gel matrix and the influence of the volumetric fraction of the dispersed particles ($\phi_{f}$, from 0 to 0.4) on the dynamic modulus of the filled gels. Gels were produced by acidification (casein gels and poly(vinyl alcohol)–Congo red gels) and filled by emulsion particles stabilized with different macromolecules. According to the results obtained, if particles did not interact with the gel matrix, the elastic modulus decreased with $\phi_{f}$. However, if particles interacted with the gel matrix, the elastic modulus was directly proportional to the value of $\phi_{f}$. Such a result was higher for acid milk gels than for PVA (polyvinylalcohol) gels, probably because the gelation step can promoted aggregation of dispersed particles. 

Generally, for active particles, the higher the volume fraction, the higher the gel strength is observed. Active filler promoted higher influence for weaker gel matrices and lower for inactive fillers [37].

Regarding the influence of the gelling material concentration and the emulsion droplet average size on the rheological properties of emulsion-filled gels, a study conducted by Sala et al. [21] investigated the effects of both parameters on large deformation properties of different gels matrices filled by emulsions stabilized with different emulsifying agents. The authors found that denser gels were formed when the gelling agent concentration was increased, creating more bonds among structural elements. Therefore, an increase in the values of Young’s modulus (E) and stress fracture was observed. As for the influence of the average droplet size, it was observed that E increased and fracture strain decreased on a decrease in the droplet size in the gels with bound and non-aggregated droplets (whey protein isolate (WPI) filled gels and gelatin gels filled by droplets stabilized by WPI). In these systems, the decrease of oil droplets size did not affect fracture stress. 

According to Dickinson and Chen [37], when there is an average droplet size reduction in emulsions stabilized by protein or surfactants of the same oil fraction contents, the interfacial area is higher along with the number of dispersed droplets. There is a balance between absorbed protein versus non-adsorbed proteins when a higher interfacial is present. Therefore, there is a tendency to change the balance between the viscous modulus (G″) and the elastic modulus (G′) of the gel matrix. However, a higher amount of dispersed droplets can promote a different behavior of the gel strength (increase or decrease), depending on the filler particle behavior (active or inactive).

Mao et al. [47] studied the influence of protein concentration (WPI; 4%–6%) and oil content (sunflower oil; 5%–20%) in emulsion-filled gels produced by cold-set gelation (glucono-δ-lactone). Higher WPI concentrations promoted an increase on the elastic modulus, G′, and a higher force and strain at breaking due to a more compact gel matrix. More rigid protein gel chains are closer, and there is a higher distribution of applied force, thus reducing breaking down of the gel matrix. With higher oil concentrations in emulsion-filled gels, filler droplets act as active fillers. A higher G′ and force at breaking were obtained for emulsion-filled gels with higher oil contents.

For emulsion-filled gels, a possible theoretical explanation about how the gel force at breaking increases with increase of oil content is due to the fact that viscous flow arises because of local yielding in the area where the stress is concentrated, thereby promoting energy dissipation. The nature of oil droplet interfacial area is viscous and can cause energy dissipation. Therefore, the force to break the emulsion-filled gels with a high oil content is higher [16,47].

In addition, the physical state of the lipid core of the emulsion droplets can influence the textural properties of emulsion-filled gels [6]. For active particles, the droplet stiffness (deformability) and consequently, their crystallinity or hardness (for solid particles) influences the rheological behavior of filled gels. However, for liquid oil droplets, the size and surface properties determine the droplet deformability and consequently, its influence on the gelled matrix [44].

Oliver, Scholten, and van Aken [6] investigated the influence of the replacement of saturated solid fat (animal fats) by unsaturated liquid oil (vegetal oils) on the textural properties (measured by uniaxial compression tests) of emulsion-filled gels. Gelled networks were produced by whey protein isolate or gelatin or micellar casein. For emulsion preparation, the emulsifier agents chosen were whey protein aggregate, whey protein, and sodium caseinate. The temperature and type of fat determined the fat hardness. The authors observed that the presence of emulsion droplets affected the fracture properties of gels, probably due to droplet distribution in the system and their stiffness (for active fillers, E is higher for droplets than for the gel matrix). The gel stiffness increased with emulsion droplets (either liquid or solid) in relation to gel with no oil added. The fat hardness had a small effect in all gels investigated. The stiffness of the whey protein aggregate gels was slightly affected by the higher content of solid fat; however, a higher increase of stiffness was observed for gelatin and micellar casein emulsion gels. Table 1 represents other studies investigating the effect of some different parameters on the emulsion-filled gel behavior. 

## 3. Rheological Techniques for the Characterization of Emulsion-Filled Gels

### 3.1. Dynamic Oscillatory Tests 

Dynamic oscillatory shear tests are often used in rheological studies to investigate complex fluids. Small amplitude oscillatory shear (SAOS) tests are methods used to study the linear viscoelastic properties of soft materials and are supported by a strong theoretical basis [57,58]. The elastic (G′), the viscous (G″) moduli, and the phase angle (δ) are the parameters studied in the characterization of the linear viscoelasticity of emulsion-filled gels. The value of G′ reflects the elastic behavior of the material tested and is represented as the energy stored, whereas G″ is associated with the viscous behavior of the material tested and refers to the amount of energy dissipated [59]. The phase angle corresponds to the arc tangent of the ratio G″/G′, and it is a measurement of the response delay of strain to the applied stress; it also indicates the relative importance of the viscous and elastic elements in the emulsion-filled gels [60].

**Figure 3:**
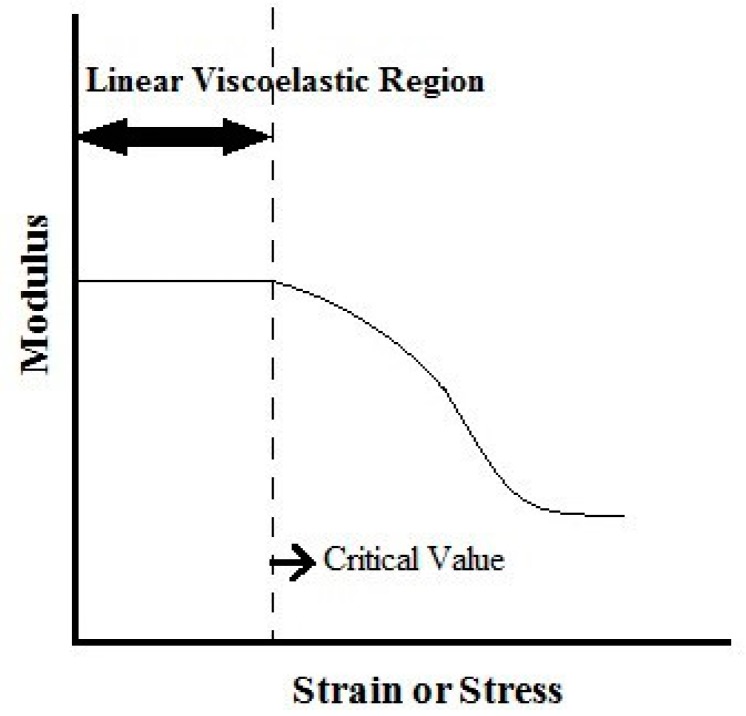
Schematic representation of the dependence of the dynamic (G′ and G″) moduli with the strain or stress and the critical value given by linear viscoelasticity limits.

Typical (SAOS) tests are divided into strain or stress sweep, frequency sweep, isothermal time sweep, and temperature sweep. A strain or stress sweep (Figure 3), is conducted by varying the amplitude of the input signal at a constant frequency and temperature. It is used to define the limits of linear viscoelasticity, indicating the limit of the strain or stress value that supports the material without occurrence of breaks or changes in its structure. In this region, the rheological properties are independent of the strain or stress [61] and SAOS tests should be performed for stresses or strains lower than their critical values. 

In the frequency sweep experiments, it is possible to observe how the viscous and elastic behavior of the material changes. In this test, the amplitude of input signal and temperature are held constant while the frequency is increased. The materials usually exhibit higher solid-like characters at higher frequencies. In strong gels, the moduli are practically independent of the frequency and do not intersect, as *G*′ > *G*″. However, when the test is conducted with weak gels, frequency dependence occurs [61]. Yang et al. [54] observed the frequency dependence of soy protein isolate and soy oil emulsion-filled gels produced by an enzymatic process and containing different oil volume fractions. They observed that for all oil volume fractions, the moduli were practically frequency independent, a typical behavior of enzymatic or strong gels due to their permanent covalent cross-links.

Regarding isothermal time sweep measurements, the frequency and the input strain, amplitudes are held constant over time and are observed during the test. In this test, the time sweep can be performed in conjunction with controlled changes in temperature [61]. In assays for the characterization of the gelation process in situ*,* the gelation of the biopolymer solutions is studied under time sweep or temperature ramp to define the time/temperature crossover, which is the intersection between the storage and loss moduli [25]. This crossover point of the moduli takes place when *G*′ = *G*″ and indicates the gel point, which is the time/temperature at which the process of gel formation starts, or the point at which *G*′ becomes larger than *G*″, with predominantly elastic characteristics [61].

Line et al. [33] observed the *G*′, *G*″, and δ behavior over time during the cold-set formation in situ of β-lactoglobulin and sunflower oil emulsion-filled gels by varying the oil concentrations. Kim et al. [23] observed small deformation oscillatory shear properties of the heat-set emulsion-filled gels formed in situ from soybean protein isolate and soybean oil and investigated the influence of different volume fractions. Both studies observed similar behaviors *G*′ > *G*″, with formation of elastic emulsion gels and a decrease in (tan δ) value over time and with the increase of temperature, respectively, which indicates the transition from a liquid-like dispersion into a more solid-like gel structure. Regarding the volume fraction, both studies concluded that the higher the oil concentration, the higher the value of *G*′, indicating that the dispersed oil droplets acted as active fillers interacting with the gel matrix.

Chen and Dickinson [17] compared in situ production of whey protein isolate gels and emulsion-filled-gels, with whey protein isolate as droplet surfactant with both gels containing the same concentration of protein. They observed that the *G*′ of the emulsion-filled gel was much higher than the *G*′ of the protein gel, which resulted in a highly active filler particle behavior of the oil droplets once they drastically increased the gel strength. Such a fact can be related to the chemical affinity between the adsorbed layers on the surface of droplets with the gel matrix, as well as the mechanical nature of the filler particle. The droplets are integrated in the structure of the emulsion-filled gels through strong covalent bonds and physical interactions between the proteins adsorbed on the surface of the droplets and the aggregated protein in the gel matrix structure and the monolayer of protein on the surface of the droplets [16].

If the surfactants used to stabilize the emulsion droplets and the gelled biopolymers are different, there is higher possibility of no affinity between them and then, the particles can act as inactive fillers due to lack of disulfide bonds, favorable hydrophobic interactions, and electrostatic interactions between the surfactant and the matrix of the gel network. In this case, the higher the volume fraction of the inactive filler, the lower the emulsion-filled gel *G*′ [16].

The strengthening of the structure occurs only if the filler particle modulus is higher than the gel modulus, and then the filler particle has a high effective *G*′ due to the Laplace pressure. *G*′ of the filler particle is proportional to the ratio between the surface tension and the droplet radius, represented by the following equation [16]:

(1) G′=2γr

Thus, it is expected that when the *G*′ of the filler particles is higher than the gel matrix modulus, the filler particles deform less and influence *G*′ of the emulsion-filled gels, and the emulsion-filled gels modulus becomes larger than the matrix modulus. This behavior was studied by Lorenzo et al. [10] in emulsion-filled gels formed with gellan gum. The obtained results showed that the *G*′ of the droplets was larger than the *G*′ of the matrix, and the strengthening of the structure of filler particles was confirmed by frequency sweep tests performed and for different oil concentrations, where the dynamic moduli were higher for higher oil fractions. However, by the van der Poel theory, with the formula extended by van Vliet (1988) to predict the shear modulus of systems containing liquid emulsion droplets, the *G*′ of filled gels depends on the relationship between the stiffness of the matrix (*G*_m_) and the stiffness of the filler particles (*G*_f_) as shown below [17,38]:

(2) $$\frac{G^{\prime }}{{G^{\prime }}_{m}}-1=\frac{15\left(1-\upsilon_{m}\right)\left(M-1\right)\phi_{f}}{\left(8-10\upsilon_{m}\right)M+7-5\upsilon_{m}-\left(8-10\upsilon_{m}\right)\left(M-1\right)\phi_{f}}$$

where *M* is the stiffness ratio (*G*′/*G*′_m_), $\phi_{f}$ is the oil fraction, and *υ*_m_ is the Poisson ratio of the gel matrix.

By applying this theory to the gellan emulsion-filled gel, Lorenzo et al. [10] observed that the reinforcement theoretical values of the emulsion-filled gels were lower than the experimental values. These differences have been explained by the fact that this theory assumes that no interaction among the particles occurs, and the particles interact only with the gel matrix [16,19]; however, in that study, the authors observed that the presence of a nonionic water-soluble emulsifier (Tween 80) allowed a weak interaction between the two systems. Therefore, the addition of oil droplets in the matrix increased the strength of the emulsion-filled gel with increasing oil fraction, but the strengthening observed by the frequency sweep test was not as expected due to the weak interaction of droplets–matrix.

Thus, as described above, this model does not take into consideration the crowding effect that occurs among the filler particles at high volume fractions. With this effect, the filler particles are aggregated and behave as particles with the volume fraction more effective than the single fillers. This effect was modeled by Lewis and Nielsen [40] by substitution of the actual volume fraction ($\phi_{f}$) (Equation (3)) by the effective volume fraction of filler as (ψ$\phi_{f}$), where:

(3) $$\psi \phi_{f}=[1+\left(\frac{1-\phi_{\max}}{\phi_{\max}^{2}}\right)\phi_{f}]\phi_{f}$$

where $\phi_{\max}$ is the maximum volume fraction of the filler particles.

Oliver et al. [20] extended this model to describe the mechanical properties of emulsion-filled gels in relation to the distribution of droplets. The model includes the effect of inhomogeneous distribution of droplets that describe the clusters, the regions with increase in volume fractions of particles dispersed on matrix that have an elastic modulus altered compared to the surrounds. The emulsion-filled gel is described as a clusters distribution, the inner phase, in the gelled matrix that also contains single fillers, the outer phase. This model assumes that the matrix is a homogenous material. Thus, the composite gel modulus is determined by the outer phase and the inner phase modules. A relative amount of single particles in the outer phase is described by a factor of correction (Ф $=\phi_{\mathrm{outer}}/\phi_{f})$ that include the ratio between the clustered and the single droplets. Thus, considering packing of the clusters, the modulus of the gel can be calculated as:

(4) $$\frac{{G^{\prime }}_{i}}{{G^{\prime }}_{\mathrm{matrix}}}=\frac{E_{i}}{E_{\mathrm{matrix}}}=\frac{15\left(1-v_{m}\right)(M_{i}-1)\psi \phi_{i}}{\left(8-10v_{m}\right)M_{i}+7-5v_{m}-\left(8-10v_{m}\right)(M_{i}-1)\psi \phi_{i}}+1$$

where *i* is inner or cluster, and

(5) $$\phi_{\mathrm{cluster}}=\frac{\phi_{f}-Ф\phi_{f}}{\phi_{\mathrm{inner}}-Ф\phi_{f}}$$

and, $M_{\mathrm{cluster}}=\frac{{G^{\prime }}_{\mathrm{inner}}}{{G^{\prime }}_{\mathrm{outer}}}=\frac{E_{\mathrm{inner}}}{E_{\mathrm{outer}}}$, $M_{\mathrm{inner}}=\frac{E_{f}}{E_{m}}$, and ${G^{\prime }}_{\mathrm{outer}}$ is calculated as:

(6) $$\frac{{G^{\prime }}_{\mathrm{outer}}}{{G^{\prime }}_{\mathrm{matrix}}}=\frac{E_{\mathrm{outer}}}{E_{\mathrm{matrix}}}=\frac{15\left(1-v_{m}\right)(M_{\mathrm{outer}}-1)Ф\phi_{i}}{\left(8-10v_{m}\right)M_{\mathrm{outer}}+7-5v_{m}-\left(8-10v_{m}\right)(M_{\mathrm{outer}}-1)Ф\phi_{i}}+1$$

In general, the model that considers an inhomogeneous distribution of droplets presents a greater increase in gel stiffness than the model that considers a homogenous distribution. 

### 3.2. Creep-Recovery Tests

The study of viscoelastic properties of multicomponent systems such as emulsion-filled gels is interesting for developing new products, in the engineering design of continuous processes, and in establishing quality control mechanisms [62].

The viscoelastic behavior of complex systems is primarily determined by oscillatory rheological measurements [63], but the internal structure of a system and changes in its structure caused by modification of its composition can also be analyzed by creep and recovery studies. Creep and recovery tests consist in deforming a viscoelastic material for a specific period of time, under a constant shear stress in either the linear or non-lineal viscoelastic regions of a material, and to measure the deformation per unit of stress (compliance–J) as function of time (creep step). Thereafter, removing the applied stress and the value of the deformation over a similar pre-established period of time is measured (recovery step) [10,61,63,64].

**Figure 4:**
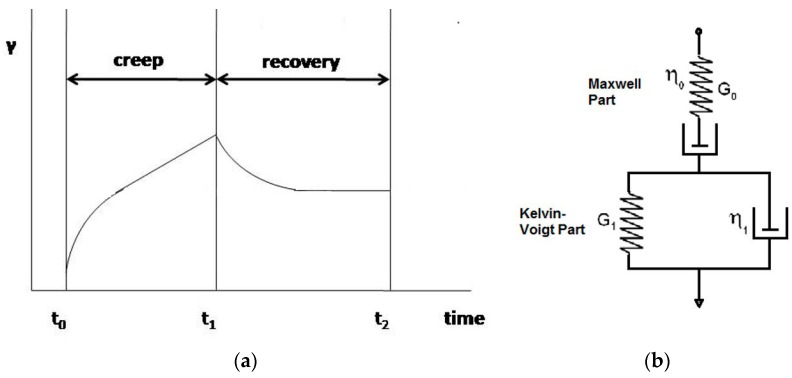
(**a**) Typical curve strain (γ) in function of time creep-recovery test for a viscoelastic material and (**b**) the four elements of Burger’s model.

Figure 4 presents a typical curve strain (γ) in function of time creep-recovery test for a viscoelastic material. 

**Figure 5:**
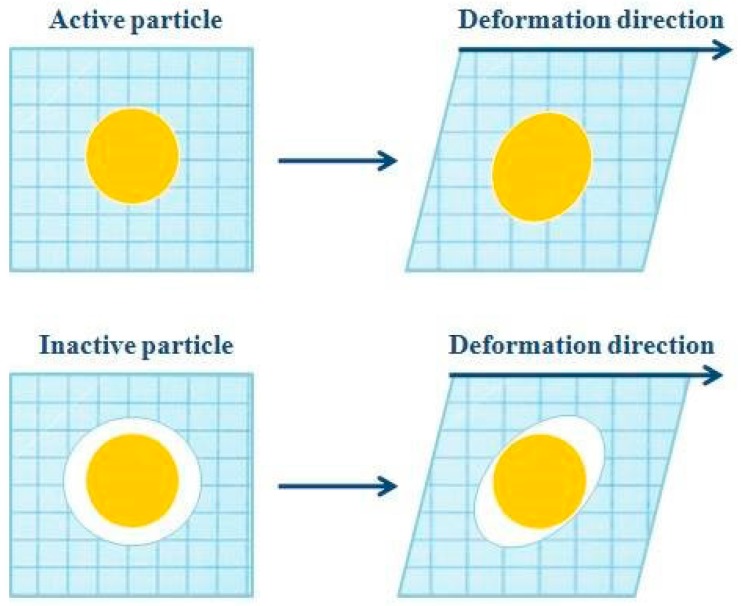
Schematic representation of behavior of an emulsion-filled gel when a shear stress is applied on active or inactive particles.

Mechanical models are used in creep-recovery tests to describe the deformation behavior of a system. A spring is used to represent purely elastic behaviors and a dashpot is used to represent purely viscous behavior. Models of Maxwell (association of a spring and a dashpot in series) and Kelvin–Voigt (association of a spring and a dashpot in parallel) are used to generalize approaches, including the association of several components. Burger’s model consists of four components (association of Maxwell and Kelvin–Voigt models) (Figure 5) and reproduces the deformation of viscoelastic material reasonably well [63]. A more detailed mathematical explanation of Burger’s model can be seen elsewhere [61].

Few studies in the literature have focused on creep-recovery analysis of emulsion-filled gels. Lorenzo et al. [64] studied emulsion-filled gels stabilized by bovine gelatin with low lipid content. The authors applied Burger’s model to fit their creep-compliance data and the model fitted well (*R*^2^ > 0.96) to the creep data.

Lorenzo et al. [10] evaluated the rheological properties of emulsion-filled high acyl gellan gum gels and investigated the effects of the gum and oil concentrations on gels. According to the authors, Burger’s model fitted satisfactorily (*R*^2^ = 0.89) the experimental data for all samples. Increasing gum concentration decreased J (compliance) due to an increase of network stiffness. Regarding the oil concentration, the values of J decreased in comparison with gels without oil, and then the oil droplets promoted reinforcement on the gel matrix [11]. Moreover, the linear viscoelastic behavior of continuous phase (gellan gum phase) and emulsion was represented by the Baumgaertel–Schausberger–Winter spectrum model. This approach showed that the continuous phase was responsible for controlling the rheological behavior of gels. 

### 3.3. Large Deformation Tests and the Correlation of Emulsion-Filled Gel Textural Properties and Sensory Perception

The textural properties of foods are one of the primary parameters considered by consumers [65] and an important quality parameter to be considered for the development of soft-solid foods such as emulsion-filled gels. This is because these properties are able to explain the behavior of emulsion-filled gels during digestion steps such as mastication [8,21,22,66].

Different mechanical processes occur during mastication, like biting, chewing, and swallowing [67]. Emulsion-filled gels are broken down in the mouth, and the tongue and the palate are responsible for exerting compressive, extensional, and shear forces to disintegrate the gel [68]. The small deformation oscillatory shear (SAOS) presents limitations to describe food textural quality [8]. The large deformation test provides important parameters to understand the mechanical properties of gels that may be involved with their consumption. For food gels, uniaxial experiments such as compression tests, as well as force and height data are converted into Hencky’s stress (σ_H_) and Hencky’s strain (ε_H_) and a curve is plotted. This curve provides gel stiffness data, represented by the modulus E obtained by the slope of a linear part of the curve. The rupture properties (stress (σ_rup_) and strain (ε_rup_)) represent the fracture point obtained by the peak of the stress-strain curve. E and fracture properties are frequently correlated with behavior of foods during mastication [69,70,71].

The large deformation experiments and fracture behavior are highly dependent on the food gel characteristics, such as the inhomogeneous features of the network structure [72]. The type of protein network and its concentration, processes of gel formation, and temperature are parameters that have a great influence on the changes in the structure of gels caused by large deformation experiments [46,73]. Moreover, gel structure and strength of the bonds in gels further determines the way in which they fracture [22].

The emulsion-filled gel modulus is affected by the presence of droplets and its dependence on the way in which the matrix and droplets interact (active or inactive particles, oil type, oil droplets size, and content) [22,46].

Sala et al. [21] described how the oil droplet size (oil droplet aggregation) can influence E of an emulsion-filled gel according to the van der Poel theory. For gels where oil droplets were active and non-aggregated, it was observed that these gels become stiffer and E increases when the oil droplet size decreases, in comparison with gels with larger droplets at the same volumetric fraction (ϕ). Different effects were observed for gels with non-aggregated and inactive oil droplets, in which the modulus E did not change. However, for gels where oil droplets were active and non-aggregated, the authors described that two effects can occur: (i) oil droplets can aggregate leading to an increase of E due to the increase of the effective volume of the droplets; and (ii) when E of the gel matrix is larger than E of the aggregates, the effect of oil droplet aggregation causes a decrease in E [21]. For emulsion-filled gels, the oil droplet aggregation affects E but has no influence on fracture properties [21].

Liu et al. [55] studied the effect on large deformation behavior (uniaxial compression) of gelatin gels filled with different fat droplets (animal and medium-chain triglycerides) stabilized by WPI (active droplets) or Tween 20 (inactive droplets). According to the authors, the E of gels increased with increase in fat content for all types of gels due to the fat droplets that were mechanically bound to the matrix (emulsions stabilized by WPI). When solid fat content increased (increase of filler E), E of the system also increased. For gels filled with droplets stabilized by Tween 20 (inactive droplets), and for fat droplets with relatively low solid fat content, E decreased with increase in fat content, according to the van der Poel theory. For inactive droplets, a small deformation just to deform an intermediate aqueous layer present between the droplets and matrix and small deformation is not able to deform droplets [16], due to the structural defects created by the aqueous layer in the gel matrix acting as “structure breakers” [17,74]. The effect of inactive droplets with high solid fat content resulted in an increase in E with increase in fat content, probably due to the contribution of crystal droplets inside the gel matrix network structure reinforcing it.

Regarding fracture, when a material is deformed, the energy necessary to deform this material (W) is related to the energy stored (W′), the energy lost (W′′_v_—by viscous flow, W′′_c_—by friction between the components and W_f_—fracture energy) (Equation (7)). 

The fracture starts in the weaker regions of the gel, such as the tip of cracks and weak spots, which act as “stress concentration” [6,22,75].

(7) $$W=W^{\prime }+{W^{\prime \prime }}_{v}+{W^{\prime \prime }}_{c}+W_{f}$$

Sala et al. [22] described that when the volume fraction (ϕ) of emulsion/oil droplets in the gelled system increased the number of structural defects also increased. Next, under deformation stress, for active particles, there was a stress concentration between the gel and the particles, breaking its interaction and resulting in fracture in the gel matrix. Therefore, there is a decrease of fracture strain and stress with increase in ϕ. However, for inactive particles, fracture occurs in the droplet gel matrix interface and both fracture strain and fracture stress decrease with increase in ϕ but with a rate smaller than that observed with active particles. Figure 5 represents a schematic representation of the situation previously discussed:

In the literature, it is possible to find some studies focusing on correlating textural perception of emulsion-filled gels with their large deformation rheological properties [19,46,65,76,77,78,79,80,81,82].

As to the other properties previously discussed, sensory aspects of the emulsion-filled gels are affected by the concentration of emulsion droplets and their size, biopolymer matrix characteristics, and the type of the interaction between matrix–droplet [82].

In emulsion-filled gels, the E and fracture properties have an important impact on the sensorial properties of the systems [79]. In the first phase of oral processing sensory properties of the products are gel firmness (E) and brittleness and these attributes, dependent on fracture or yield stress and on apparent modulus, are measured at large deformation [70,83,84]. In another study, Çakir et al. [85] correlated E to the perceived hardness of gels and the fracture stress was correlated with hardness perception of cheese [86,87]. De Lavergne et al. [65] investigated how the composition of emulsion-filled gels and how their fracture properties can influence the dynamic textural perception of the systems. Gels with different fracture properties (fracture stress and fracture strain) were prepared by changing the content of gelatin and polysaccharides (agar, κ-carrageenan, locust bean, and high/low acyl gellan) mixture. The authors observed that all gels with different compositions exhibited similar fracture properties. The perception of texture is influenced by the fracture stress in the first bit and by the fracture strain during chew down. Creamy gels were obtained at high fracture strain and higher chewing force is necessary for gels with a high fracture stress.

In another study, de Lavergne et al. [26,80] correlated fracture properties to the attributes toughness and elasticity (high fracture stress and strain); lumpy and grainy (high fracture stress and low fracture strain), and perception of creaminess (low fracture stress and high fracture strain) in emulsion-filled gels.

Making use of another approach, Paradiso et al. [11] studying inulin emulsion-filled gels produced with different oil contents (high, medium and low) used penetration tests to measure the consistency of the gels and correlated the results with the consistency perceived by the panelists in sensorial analysis. The authors found that the consistency measured by the penetration test increased with decrease in oil content in gels (gels with inactive particles). The sensorial evaluation proved that the samples with low and medium oil content were perceived as more consistent than samples with high oil content, by sensory evaluation.

Another approach has been investigated in the past few years which correlates large deformation rheology measurements and simultaneous microscopic observations to understand the effect of structural properties of multicomponent foods such as emulsion-filled gels in mastication [88,89].

Abhyankar et al. [88] studied the impact of notch propagation tensile testing on large deformation properties of heat-set whey protein (WP) gels with sodium caseinate (NaCN) or sunflower oil droplets (WP or NaCN used as emulsifier) at an ionic concentration of 0 or 50 mM NaCl. The microstructural changes were observed by confocal laser scanning microscopy (CLSM). The parameters NaCl concentration, oil concentration, oil droplet aggregation and emulsifier type impacted on the large deformation measuring properties of the gelled systems studied. The structural properties of the gel matrix as well as proximity of the oil droplet to the fracture path influenced tensile testing as demonstrated by the CLSM images.

In another study, Abhyankar et al. [89] combined large deformation properties of emulsion-filled gels (heat-induced whey protein gels; pH 7.0 or 5.4 with emulsified sunflower oil) with CSLM analysis to observe microstructural properties changes of the system upon deformation. To this end, the authors monitored the fracture behavior and microstructure of gels in extension using microtensile stretching. For emulsion-filled gels at pH 5.4 the modulus E was higher, the critical fracture stress was higher, and the fracture strain was lower than the ones measured for gels at pH 7. A homogeneous protein phase of gel prepared at pH 7, with fat droplet-entrapped and droplets from spherical to elliptical shape during deformation, was observed by CLSM. The authors suggested that in this gel (pH 7), having droplets acted as active fillers. However, the opposite was observed by CLSM for the gel prepared at pH 5.4: a non-homogeneous protein phase (porous) and droplets appeared disconnected from the protein matrix, with formation of pools due to oil droplet coalescence/breakage, like typical behavior of inactive droplets. Moreover, in the gel prepared at pH 7, fracture occurred through droplets, releasing free fat, while for gels produced at pH 5.4, the droplets behaved as inactive particles and were free to move and coalesce.

It can be observed in these studies that a good understanding about the relationship between sensorial, rheological, physicochemical, and (micro) structural properties of emulsion-filled gels can facilitate the development and application of these systems in modern food production [19].

## 4. Conclusions and Future Perspectives

Several current food products such as yogurt, ice cream, cheese, and processed meat products can be classified as emulsion-filled gels.

The replacement of fat while maintaining quality attributes is a need in these foods and is a challenge to the food industry. Several biopolymeric ingredients such as proteins and polysaccharides can be used to replace fat due to their interesting functionality, such as gelling agents that improve structural properties. The rheological study concerning the impact of the replacement of ingredients in soft-solid food is crucial to guarantee their quality and adequate processing methods.

In this review, we discussed the studies that have been conducted in recent years on emulsion-filled gels and how rheology studies have contributed in understanding the characteristics of these kind of materials. We noticed a range of different rheological behaviors, and changes of textural attributes of emulsion-filled gels can be obtained solely by manipulating certain parameters as follows:

(i) different biopolymers as gelling agents;
(ii) oil type and content and its physicochemical properties;
(iii) interaction between emulsion droplets and biopolymeric matrix;
(iv) production processes.

Moreover, the different rheological approaches used to study these systems have been very useful in understanding the textural properties and their influence on sensorial perception. This knowledge is extremely important in improving the quality of these products and ensure consumer satisfaction.

Because consumers demand healthier foods, as in the near future with emulsion-filled gel research, we can expect encapsulation techniques associated with emulsion-filled gel production. Some recent studies have been published regarding the incorporation of bioactive compounds into emulsion-filled gels and how these bioactive components are processed under gastrointestinal conditions. Therefore, rheological characterization of these new food products will be an important tool for this purpose.

## Figures and Tables

**Table 1:** Some interesting rheological studies on emulsion-filled gels found in the literature.

References	Effect Studied	Principal Results
[48]	Droplet size and emulsifier agent type on the rheology of WPI emulsion-filled gels	Gel strength of emulsion stabilized by WPI was higher than gel strength of emulsion stabilized by nonionic surfactants and gel strength of emulsion stabilized by sodium dodecyl sulfate (SDS). For droplets stabilized by WPI, the gel strength increased as the droplet size decreased. A relatively insensitive effect on the gel strength was observed for droplets stabilized by small-molecule surfactants.
[49]	Influence of nonionic emulsifier (Tween 20) on rheological behavior of β-lactoglobulin emulsion gels	G′ (storage modulus) demonstrated increase at low emulsifier content, decrease at intermediate emulsifier content, and then at high emulsifier contents it either increased again or remained low, depending on the protein content.
[23]	Volume fraction of oil droplets in soybean protein gels	Increasing oil volume fraction, storage and loss moduli (G′ and G″) of the gels increased. Higher compressive stresses of the gels containing smaller oil droplets were obtained in comparison to those containing larger droplets, especially at higher volume fractions of oil droplets.
[33]	The influence of oil and calcium concentrations on the rheological properties of cold gelation of β-lactoglobulin emulsion gels	The oil content affected G′ (storage modulus) of gels, the effect of which was higher than that of calcium concentration.
[50]	Emulsion droplets size influence and fat content influence in preheated emulsions stabilized by whey protein produced by cold gelation	The storage modulus (G′) of the emulsion-filled gels increased with decreasing emulsion droplets size and increasing fat content.
[51]	Different types of oils in GDL-induced SPI gels	The SPI gels filled by palm stearin emulsion were harder than the SPI gels filled by soy and sunflower oil emulsions as well as the SPI gel without oil. Gels containing the two liquid oils were softer than the control (SPI gel without oil).
[52]	Emulsions of vegetable oils (olive and peanut) of various particle sizes in composite gels with 2% myofibrillar protein	Increases in storage modulus (G′) of myofibrillar protein sols/gels with the addition of emulsions. G′ increases with smaller emulsion droplet size. Hardness of gels containing olive oil emulsion was higher than that containing peanut oil emulsion. Myofibrillar protein as an emulsifier agent promoted stronger reinforcement of the gels than the oil droplets stabilized by Tween 80-stablized oil droplets.
[53]	Presence of emulsified olive oil effect on G′ of gelatin-starch phase-separated gels	The dispersed oil phase behaved as an active filler within the phase-separated gel matrix due to increase of G′ after oil incorporation in the gels.
[54]	Different oil fractions in SPI-stabilized emulsion gels	Increase in oil fraction progressively increased the storage modulus (G′) (gel strength).
[10]	Oil and gellan gum concentrations on the viscoelastic behavior of high acyl gellan gumemulsion-filled gels	Stronger gels were obtained with increasing gellan concentration, but oil fraction had a small effect on the elastic behavior of the emulsions.
[55]	Droplet–matrix interactions in gelatin gels filled by O/W animal fat or oil emulsions stabilized with different emulsifiers	The mechanical properties of the gels (Young’s modulus (E) and fracture properties) were affected by droplet–matrix interaction, fat content, and solid fat content.
[56]	The volume fraction of soybean oil the rheological behavior of acid- and salt-induced soft tofu-type gels	A lower gel point temperature and a reduction in gelation time were observed for a high oil volume fraction, while an increase was observed in the storage modulus at the gel point when the oil volume fraction in the gels increased.
